# Plasma Cytokine Profiles in Long-Term Strenuous Exercise

**DOI:** 10.1155/2016/7186137

**Published:** 2016-04-28

**Authors:** Hilde G. Nielsen, Olav Øktedalen, Per-Kristian Opstad, Torstein Lyberg

**Affiliations:** ^1^Division for Society and Health, Department of Health, The Research Council of Norway, P.O. Box 564, 1327 Lysaker, Norway; ^2^Department of Infectious Medicine, Oslo University Hospital, Ullevål, 0407 Oslo, Norway; ^3^Norwegian Research Defence Establishment, P.O. Box 25, 2027 Kjeller, Norway; ^4^Norwegian Military Academy, 0593 Oslo, Norway; ^5^Department of Medical Biochemistry, Oslo University Hospital, Ullevål, P.O. Box 4956, Nydalen, 0424 Oslo, Norway

## Abstract

The open window theory indicates altered immunity 3 to 72 hours after exercise. The J-curve describes the risk of illness in response to exercise. The aim of this study was to examine the secretion of proinflammatory and anti-inflammatory cytokines before and after long-term strenuous exercise. Fourteen marathon and 16 half-marathon runners and 10 military cadets participating in a military ranger-training course were recruited to this study. Within-subject design was used measuring levels of plasma cytokines before, during, and after exercise. Plasma cytokines were measured using Luminex multiplex technology and ELISA. Comparing pre/post plasma levels both the marathon- and the half-marathon runners showed heavily increased levels of IL-6, IL-10, and IL-8 (*P* < 0.001). LPS stimulation among the half-marathon runners decreased the postrace levels of IL-6, IL-1b, and TNF*α* by 45%, 24%, and 43%, respectively (*P* < 0.01). During the ranger training course the spontaneous and LPS-stimulated levels of IL-6, IL-8, IL-10, IL-1b, and TNF*α* changed in a similar fashion as in the half-marathon runners although the fluctuations were smaller. Our study supports the open window and the J-curve theory; the immune system is more activated and the subjects are more threatened to infectious pathogens after intensive physical activity and in the period after exercise.

## 1. Introduction

The open window theory implies that there is altered immunity following an acute bout of exercise, which lasts from three to 72 hours after exercise depending on the parameter measured [1]. According to the theory, there is an increased risk of contracting infectious diseases after physical exercise [2]. The J-curve describes the risk of illness in response to exercise. The “J” means that individuals engaged in moderate physical activity are at lower risk compared with sedentary individuals [3]. On the other side, excessive volumes of strenuous endurance exercise may suppress immune function and thereby enhance the risk of illness [2].

Inflammation is a complex series of events, involving many cell types. It is evident that great numbers of molecules encompass the term “proinflammatory cytokines.” These cytokines are produced very early in the response to multiple stresses and are important by being involved in both innate and acquired immunity. They function as intercellular signals that regulate local and systemic responses. Cytokines are characterised as being inducible and belong to different families, including proinflammatory cytokines (IL-1*β*, IL-2, IL-4, IL-6, IL-8, and TNF*α*), which are cytokines that promote systemic inflammation, and anti-inflammatory cytokines (IL-5, IL-6, and IL-10), which refer to the property of a substance that reduces and protects against inflammation. Inflammation in excess is detrimental and excessive production and release of TNF*α* and IL-1*β* may lead to pathology [4].

Vascular endothelial growth factor (VEGF) is a signal protein produced by cells that stimulates vasculogenesis and angiogenesis. VEGF participates in restoring oxygen supply to tissues when blood circulation is insufficient. Proinflammatory cytokines like IL-1*β*, IL-6, IL-8, TGF-*β*, and TNF-*α* variably increase VEGF expression, depending upon the dose, cell, or tissue type. When VEGF is overexpressed, it can contribute to disease [5].

Regular exercise and its effects may be a prototype of physical stress [6]. Surgery, trauma, burn, and sepsis induce a pattern of hormonal and immunological responses with the same response profile as that of exercise [7]. Earlier studies regarding exercise and effects on immunological parameters indicate that the profiles of proinflammatory and anti-inflammatory cytokine levels are stimulated based on the intensity and duration of the physical exercise [8, 9]. Long-distance runners, for example, marathon runners, have shown high postexercise levels of IL-1*β*, IL-6, and IL-8 after six hours' endurance exercise [9–11], while there were no changes observed in plasma cytokine levels after a 5-kilometre run [12]. Measurements from cycle ergometer and treadmill running show no response of short-term exercise with high and moderate intensity, an increase in IL-6 after one-hour cycling at 75% of maximal oxygen consumption (VO_2_max) and three-hours alternating cycling and treadmill running at 60–65% of VO_2_max showed high postexercise values of IL-6, IL-1*β*, and TNF*α* [13–15]. Other studies indicate that long-term sleep and energy deficiency lead to immune depression [16, 17].

Microsphere-based multiplexing bioassay system has, during the last few years, become an important tool in cytokine detection. The assay is performed with great speed and accuracy by making use of hundreds of specially prepared magnetic beads, or microspheres, and is reporting on the contents of the sample. This is in contrast to the classical analysis of cytokine expression patterns, which has been performed by enzyme-linked immunosorbent assays (ELISA) for each separate analysis. Very few studies within the field of exercise immunology have so far used the multiplex technology in cytokine detection in relation to exercise or physical activity executed by healthy people.

According to prior reports, we expect in our study that the marathon/half-marathon race and the ranger-training course will lead to immunological responses in the subjects as seen in surgery and sepsis patients.

The aim of this study was to examine the secretion of the cytokines IL-1*β*, IL-2, IL-4, IL-5, IL-6, IL-8, IL-10, IL-12, TNF*α*, IFN_*γ*_, GM-CSF, and VEGF before and after three different trials of physical exercise: the Oslo marathon/half-marathon and a military training course of eight days continuous exercise combined with sleep and energy deprivation. Both the spontaneous secretion of the cytokines and the lipopolysaccharide- (LPS-) induced whole blood stimulation of cytokines were examined.

## 2. Material and Methods

### 2.1. Oslo Marathon/Half-Marathon and Norwegian Military Ranger-Training Course

Fourteen men participating in Oslo marathon race (mean age 40; range 29–56), eight women (mean age 36; range 27–39) and eight men (mean age 34; range 30–45) participating in Oslo half-marathon race, and ten physically well-trained male cadets from the Norwegian Military Academy in the age between 21 and 28 years were recruited to this study. The marathon runners were selected based on the previous year's result list with the criterion that the expected running time should be 3 h 30 min; the corresponding criterion for the half-marathon runners was 1 h 30 min for men and 1 h 45 min for women. The runners were on average fit and practiced running as an interest. The race was conducted in cloudy weather with a temperature of about 20°C. The ranger-training course took place in the eastern part of Norway, in a forest area at 500 m altitude. The temperature during the eight days of the course was between 18 and 30°C during daytime and between 5 and 15°C at night. The ranger-training course consisted of military duties during daytime and nighttime [16]. The cadets were provided nearly no food during the course and approximately 3 hours/8 d sleep was allowed [16]. In the restitution period (day 9 through day 11), the cadets followed regular sleeping and eating habits as before the course. The subjects were informed about the study and gave their written informed consent for participation. The Regional Committee for Medical Ethics had approved the test protocols.

### 2.2. Blood Sampling Protocols

For the marathon runners and the half-marathon runners, blood samples were collected shortly before the race and immediately after the race. For the military cadets, blood samples were collected at the start of the ranger-training course, after 2, 4, and 8 days (end of the ranger-training course), and after one (day 9) and three (day 11) days' recovery. The blood samples from the cadets were taken between 6 and 8 a.m. For all groups, venous blood was sampled into either EDTA-, Na-citrate-, or heparin-anticoagulated vacuum tubes (Becton Dickinson, Plymouth, UK). The blood samples from the ranger-training course were collected in the field, kept on ice, and transported within 60 minutes to the nearest military training camp, Terningmoen, where a temporary laboratory was established. The blood samples from the marathon and the half-marathon runners where collected in the start area of the race, kept on ice, and transported within 30 minutes to Ullevål university hospital. Plasma was separated at 2500 ×g for 10 minutes and samples were stored at −80°C until analysis. To compare the cytokine production capacities of leukocytes, one set of experiments was performed with heparinized whole blood from the half-marathon runners and the ranger-training cadets. The blood samples were stimulated* ex vivo* with the potent cytokine inducer lipopolysaccharide (LPS), 1 *μ*g/mL (*working solution*) (from* Escherichia coli* 026: B6, Sigma St. Louis, Mo, USA) for 6 h at 37°C, before storing plasma samples at −80°C until analysis.

### 2.3. Haematological Analyses

White blood cell (WBC), platelet and erythrocyte counts, haemoglobin, and haematocrit were assessed in EDTA blood using the Technicon H2*∙™* System (Bayer Corporation, Tarrytown, NY, USA) at the Department of Medical Biochemistry, Oslo University Hospital, Ullevål.

### 2.4. Cytokine Analyses

#### 2.4.1. Individual Cytokine Assays

Plasma levels of individual cytokines (IL-1*β*, IL-6, IL-8, IL-10, and TNF*α*) were measured using the Quantikine colorimetric sandwich enzyme-linked immunosorbent assays (ELISA) from R&D, Abingdon, UK, according to the manufacturer's instructions.

#### 2.4.2. Multiplex Cytokine Assays

We used the multiplex bead-based sandwich immunoassay technology (Luminex, Austin, TX, USA) and a human cytokine 12-plex kit (R&D), strictly following the manufacturer's instructions, to measure the concentration of the following cytokines before and after the marathon race: IL-1*β*, IL-2, IL-4, IL-5, IL-6, IL-8, IL-10, IL-12, TNF*α*, IFN_*γ*_, GM-CSF, and VEGF.

Unfortunately, we only had the multiplex bead-based sandwich immunoassay technology (Luminex) available for the study with the marathon runners. The levels of cytokine release in the half-marathon runners and the cadets from the ranger-training course were therefore measured using the Quantikine colorimetric sandwich enzyme-linked immunosorbent assays (ELISA).

### 2.5. Statistical Analyses

Results are given as means and standard errors of the mean (mean, SEM). Data were analysed using one-way ANOVA with Student's *t*-test. Corrections for multiple uses of the tests were performed* ad modum* Bonferroni. *P* values of less than 0.05 were considered statistically significant. Difference within groups (marathon and half-marathon) was analysed using two-tailed paired sample *t*-test. Independent sample *t*-test was used in order to compare men and women within the half-marathon group. Since we did not observe any statistically significant differences between the women and men, we have chosen to present the results from the half-marathon runners as one group. Paired sample *t*-test was used to compare results from day 0 to results after 2, 4, and 8 days during the ranger-training course, and after day 1 and 3 days recovery. In the ranger-training course the repeated and longitudinal values obtained before, during, and after the period of exercise which made the subjects being their own controls.

## 3. Results

### 3.1. Haematology

The total leukocyte counts (WBC) (10^9^/L) increased by 3.2-fold (values before: 5.1, values after: 16.4, *P* < 0.01) after the marathon race, 2.4-fold (values before: 6.3, values after: 15.2, *P* < 0.01) after the half-marathon race, and 1.5-fold (values before: 6.3, values after: 9.5, *P* < 0.01) at the end of the ranger-training course (day 8). After the ranger-training course, WBC were back to normal values after one-day (day 9) recovery. The increase in WBC was mainly due to a significant increase in neutrophils after both of the marathon races and after the ranger-training course, but monocyte counts were also consistently increased in all three experimental settings.

The platelet counts (10^9^/L) increased significantly after both the marathon race (values before: 194, values after: 243, *P* < 0.01) and the half-marathon race (values before: 255, values after: 313, *P* < 0.05) and after eight days at the ranger-training course (values before: 214, values after: 265, *P* < 0.05). Baseline platelets values were observed after three days' recovery (day 11).

We observed no changes in haemoglobin values after the marathon and the half-marathon races. A slight decrease in haemoglobin values was observed after eight days in the military training course (*P* < 0.05), maintained during the whole recovery period. The haematocrit values remained unchanged after the running races, whereas the values were reduced during days 4–8 of the ranger-training course as well as during the recovery phase.

### 3.2. Cytokine Profiles

**Figure 1:**
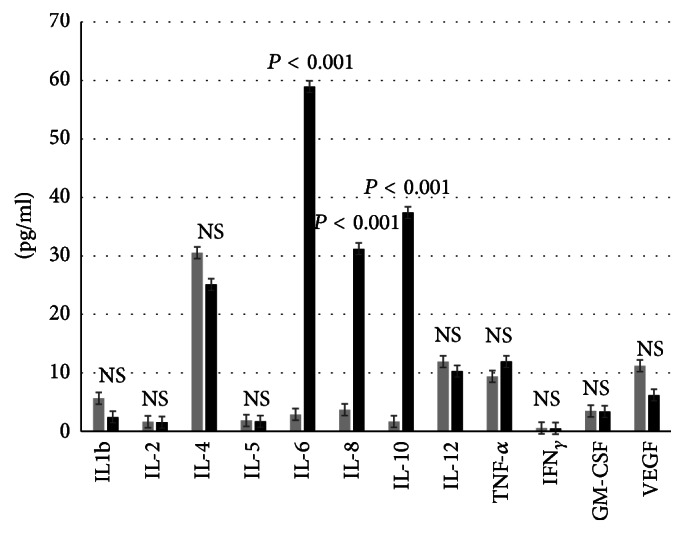
Multiplex cytokine analyses (pg/mL) before (grey colour) and after marathon race (black colour). *n* = 14. Data are expressed as mean and standard error of the mean. NS: nonsignificance.

*Marathon (Figure 1)*. We performed a plain analysis of plasma levels of 12 cytokines in 14 individuals before and after a marathon race. Three of these cytokines were heavily increased when comparing pre/post plasma levels: IL-6 (26-fold increase, *P* < 0.001), IL-10 (28-fold increase, *P* < 0.000), and IL-8 (11-fold increase, *P* < 0.001). The other measured cytokines, IL-1*β*, IL-2, IL-4, IL-5, IL-12, TNF*α*, IFN_*γ*_, GM-CSF, and VEGF, did not change significantly after the marathon race. 

*Half-Marathon (Table 1)*. In the half-marathon race we demonstrated significantly increased plasma levels of IL-6 (40-fold increase, *P* < 0.001), IL-10 (10-fold increase, *P* < 0.001), and IL-8 (14-fold increase, *P* < 0.001), whereas IL-1*β* and TNF*α* were unchanged after the race. When examining LPS-stimulated cytokine levels, IL-6, IL-1*β*, and TNF*α* were reduced after the race (IL-6 45% reduced, *P* < 0.001; IL-1*β* 24% reduced, *P* < 0.05; and TNF*α* 43% reduced, *P* < 0.001). On the contrary, IL-10 and IL-8 were further increased when LPS stimulation was superposed; IL-10 225% increased, *P* < 0.01, and IL-8 242% increased, *P* < 0.001. 

*Ranger-Training Course (Tables 2 and 3)*. The subjects of the ranger-training course showed smaller increase than in the half-marathon race with surprisingly highest levels in the postrace recovery period (Table 2). IL-1*β*, IL-6, IL-8, IL-10, and TNF*α* were all significantly increased at some stage during the course. Additional investigations including LPS stimulation of whole blood showed reduced production capacity for IL-1*β*, IL-6, and TNF*α*, whereas the IL-8 and IL-10 levels were further increased in a similar manner as after the half-marathon (Table 3).

## 4. Discussion

Several studies have observed leukocytosis after physical exercise [2, 18, 19]. Physical exercise leads to an excessive increase in total number of neutrophils immediately and is followed by a slightly smaller increase a few hours after the exercise [20]. In our study, the blood samples were taken immediately after the exercise and only the initial increase in leukocytes was measured. We observed a 3.2-fold and a 2.4-fold increase in leukocytes after the marathon race and the half-marathon race, respectively. At the end of the ranger-training course (day 8), the levels of leukocytes had increased by 1.5-fold. In agreement with former studies we found that the increase in leukocyte counts is primarily due to increase in neutrophils, although monocytes and lymphocytes were increased as well [21]. The first increase in neutrophils is mainly due to release of marginated cells caused by mechanical shear stress and catecholamines [22]. The reason for the different levels of leukocytes in the three exercise trials is probably due to the type and the duration of the activity [21], and this corresponds to earlier studies [23, 24].

Altered levels of cytokines are not only seen in inflammatory diseases. Acute exercise in healthy individuals has an effect on cytokines and the inflammatory response as well [9]. The intensity, duration, and type of physical exercise can all influence proinflammatory and anti-inflammatory cytokines and thereby influence the susceptibility to inflammatory diseases [19]. Ostrowski et al. [4, 25] reported a 2-fold increase in TNF*α* and IL-1*β* after a marathon race, in addition to a nearly 100-fold increase in IL-6 values. Exercise studies of three hours' duration performed in laboratories have shown increase in the same parameters [9].

We observed after the marathon and the half-marathon races a 26-fold and a 40-fold increase in IL-6. The magnitude of increase in IL-6 is closely related to the duration of the activity [26], the age of the athlete, the body mass index (BMI), and the physical condition of the person [13, 14]. The participants in our trials were moderate to well-trained individuals executing physical exercise of long duration. The half-marathon runners were on average 5 years younger compared to the marathon runners, they run half the distance of a marathon race, and they probably ran the half-marathon race with higher heart rate compared to the level of intensity of the marathon runners. Our study did not separately measure the effect of running intensity and running duration on the plasma cytokine levels after marathon and half-marathon running.

For the marathon runners we observed 11-fold and 28-fold increase in IL-8 and IL-10, respectively, but no change in TNF*α* and IL-1*β*. In the half-marathon runners, we observed a 10-fold increase in IL-10 and 14-fold increase in IL-8, but as for the marathon race, no changes in IL-1*β* and TNF*α* values were recorded. It seems that TNF*α* and IL-1*β* do not increase exponentially during exercise, which is different when compared to infections [27]. It could be hypothesized that the high levels of the anti-inflammatory cytokines IL-6 and IL-10 in our study might prevent the production of TNF-*α* and IL-1*β* after the races.

IL-6 is called an inflammatory responding cytokine described as a proinflammatory cytokine but can also stimulate anti-inflammatory cytokines. IL-6 is highly increased during strenuous physical exercise and thus takes part in the control of the inflammatory response to strenuous physical exercise [25, 28]. The proinflammatory TNF*α* is known to be inhibited by IL-10 [29] and induced by different stressors [30, 31]. The values obtained after physical exercise are inconstant related to the variations in duration and intensity of the exercise tasks [32].

In the ranger-training course, there was no increase in IL-6 or IL-10 during the period of activity, but, surprisingly, a small increase in the recovery phase (days 9 and 11). IL-1*β* and IL-8 showed a small increase during the course and in the recovery period. Both IL-1*β* and TNF*α* showed elevated values in the postexercise period.

The boosting of cytokine responses in peripheral blood leukocytes by LPS in many ways mimics the initial innate immune response to bacterial infection, LPS stimulation showed that the plasma levels of both IL-6, IL-1*β*, and TNF*α* were significantly reduced after the half-marathon race as well as during and after the ranger-training course. This corresponds to the observation made by Abbasi et al. [33] who have observed reduced IL-6, IL-1*β*, and TNF*α*- levels in LPS-stimulated samples 30 min and 3 h after exercise in half-marathon runners.

The observed reduction after the half-marathon race and the ranger-training course may be due to either actually reduced production capacity as a result of exercise or at least a temporary change in the regulatory pathways leading to altered cytokine synthesis and release. In contrast, IL-8 and IL-10 were boosted both by the exercise itself and after additional LPS stimulation. It seems that the anti-inflammatory IL-10 production is more triggered in the long-lasting marathon race (28-fold increase) compared with the shorter workload of the half-marathon race (10-fold increase). Furthermore, the IL-6 release was more pronounced in the more work-intensive half-marathon than the longer-lasting but less intensive marathon race (40-fold versus 26-fold increase), possibly being mainly due to a larger release from the muscle mass of the lower extremities [34]. Although a similar cytokine profile was registered in the ranger-training course, the magnitude of cytokine increases was not as impressive as for the corresponding marathon races, again possible due to a lesser work load per time unit as well as the lower work intensity. Regardless, we have in all three experimental settings demonstrated an increase of proinflammatory and anti-inflammatory cytokines, which in many ways are similar to the cytokine profile, which characterises inflammatory and infectious diseases [8, 27]. The balance between proinflammatory and anti-inflammatory cytokines during exercise may lay the ground for potentiation of the inflammatory status by introducing additional coincidental pathogens of both Gram-negative and Gram-positive origin, which thereby can tilt the balance into overt disease. Both LPS of Gram-negative bacteria and lipoteichoic acid (LTA), peptidoglycan, and purified protein derivative (PPD) of Gram-positive bacteria all have a number of biological activities, including also being potent cytokine inducers [35, 36].

Most of the studies do only look at the direct immunological responses occurring immediately after different types of physical activity. The very new part in our study is the three days follow-up after the end of the ranger-training course. The participants responded to a phone questionnaire one to two weeks after the course. None of the respondents reported upper respiratory tract infections or other infectious diseases. It may, however, be noted that the training course was performed at a season of the year where the general burden of common cold or influenza-like disease is at a minimum.

## 5. Conclusion

In conclusion, our study of long-term physical exercise performed in three different trials showed a significant increase in plasma levels of the cytokines IL-6, IL-8, and IL-10, whereas TNF*α* and IL-1*β* levels were unchanged or reduced. Our study supports the open window theory by an “open window” of altered immunity after long-term physical exercise and which lasts for at least 72 hours after the exercise. The results support the J-curve theory; athletes are more sensitive to pathogens in the immediate period after intensive physical exercise, probably due to the exercise-induced inflammatory state that can exaggerate the response to coincidentally occurring pathogens.

## Figures and Tables

**Table 1:** Individual cytokine analyses before and after half-marathon race. *n* = 16.

Cytokine	Unstimulated samples (pg/mL)			LPS-stimulated samples (ng/mL)		
	Pre	Post	*P* value	Pre	Post	*P* value
IL-1*β*	0.40 ± 0.02	0.5 ± 0.13	NS	72.7 ± 8.1	56 ± 8.0	0.05
IL-6	1.1 ± 0.2	36 ± 6	0.001	74 ± 5	45.4 ± 6.2	0.001
IL-8	1.7 ± 0.1	15.5 ± 1.1	0.01	4.4 ± 0.5	9.5 ± 1.30	0.001
IL-10 (pg/mL)	8.0 ± 0.2	71.8 ± 11.1	0.001	165 ± 28	275 ± 32	0.01
TNF-*α*	7 ± 3	5.3 ± 0.1	NS	53 ± 6	28.9 ± 4.5	0.001

Data are expressed as mean and standard error of the mean. NS: nonsignificance.

**Table 2:** Individual cytokine analyses (pg/mL) before, during, and after the ranger-training course. Unstimulated samples. *n* = 10.

Unstimulated samples						
Cytokines	Day 0	Day 2	Day 4	Day 8	Day 9	Day 11
IL-1*β*	0.23 ± 0.04	0.4 ± 0.1	0.7 ± 0.2^*∗*^	0.4 ± 0.1^*∗*^	0.6 ± 0.1^§^	1.2 ± 0.2^#^
IL-6	2.2 ± 0.3	4.9 ± 1.8	4.3 ± 1.4	4.50 ± 1.02	6.6 ± 1.3^*∗*^	6.1 ± 2.2
IL-8	1.1 ± 0.1	0.7 ± 0.1	2.0 ± 0.2^*∗*^	1.5 ± 0.1	3.3 ± 0.3^*∗*^	1.5 ± 0.1
IL-10	20.8 ± 5.4	28.1 ± 6.1	21.1 ± 2.5	27.2 ± 2.8	36 ± 3^*∗*^	43.3 ± 1.5^#^
TNF-*α*	1.6 ± 0.1	1.5 ± 0.2	1.4 ± 0.2	1.5 ± 0.2	2.04 ± 0.24^*∗*^	2.1 ± 0.2^§^

Data are expressed as mean and standard error of the mean. ^*∗*^ *P* < 0.05, ^§^ *P* < 0.01, and ^#^ *P* < 0.001.

**Table 3:** Individual cytokine analyses (ng/mL) before, during, and after the ranger-training course. LPS-stimulated samples. *n* = 10.

LPS-stimulated samples						
Cytokines	Day 0	Day 2	Day 4	Day 8	Day 9	Day 11
IL-1*β*	119.6 ± 21.5	42.7 ± 4.6^§^	61.2 ± 7.4^*∗*^	50.4 ± 6.2^*∗*^	126.2 ± 22.6	67.4 ± 12.8^**#**^
IL-6	72 ± 4.5	41.1 ± 3.9^**#**^	52.5 ± 4.5^**#**^	41.9 ± 3.6^**#**^	68.1 ± 6.7	52.4 ± 4.6^**#**^
IL-8	11.1 ± 1.5	11.1 ± 1.4	17.4 ± 1.9^*∗*^	13.1 ± 1.7	10.4 ± 1.2	9.7 ± 0.7
IL-10 (pg/mL)	90 ± 15	170.1 ± 30.5^*∗*^	485.9 ± 85.5^**#**^	350 ± 90^§^	156.8 ± 41.4	90.9 ± 25.1
TNF-*α*	64.9 ± 8.2	53.2 ± 6.7	62.2 ± 7.8	60.9 ± 8.8	38.5 ± 3.6^*∗*^	53.3 ± 4.7

Data are expressed as mean and standard error of the mean. ^*∗*^ *P* < 0.05, ^§^ *P* < 0.01, and ^#^ *P* < 0.001.
